# Disease-modifying treatment and disability progression in subclasses of patients with primary progressive MS: results from the Big MS Data Network

**DOI:** 10.1136/jnnp-2024-334700

**Published:** 2024-12-06

**Authors:** Johannes Lorscheider, Alessio Signori, Suvitha Subramaniam, Pascal Benkert, Sandra Vukusic, Maria Trojano, Jan Hillert, Anna Glaser, Robert Hyde, Tim Spelman, Melinda Magyari, Frederik Elberling, Luigi Pontieri, Nils Koch-Henriksen, Per Soelberg Sørensen, Oliver Gerlach, Alexandre Prat, Marc Girard, Sara Eichau, Pierre Grammond, Dana Horakova, Cristina Ramo-Tello, Izanne Roos, Katherine Buzzard, Jeanette Lechner Scott, José Luis Sánchez-Menoyo, Raed Alroughani, Julie Prévost, Jens Kuhle, Orla Gray, Guillaume Mathey, Laure Michel, Jonathan Ciron, Jérôme De Sèze, Elisabeth Maillart, Aurelie Ruet, Pierre Labauge, Helene Zephir, Arnaud Kwiatkowski, Anneke van der Walt, Tomas Kalincik, Helmut Butzkueven, On behalf of Italian MS Register, Alessio Signori, On behalf of Observatoire Français de la Sclérose en Plaques (OFSEP), Guillaume Mathey, On behalf of MSBase Study Group, Johannes Lorscheider, On behalf of Swedish MS Registry, Jan Hillert, On behalf of Big MS Data Network, Johannes Lorscheider

**Affiliations:** 1 Department of Neurology, University Hospital Basel, Basel, Switzerland; 2 Research Center for Clinical Neuroimmunology and Neuroscience Basel (RC2NB), University of Basel, Basel, Switzerland; 3 Department of Health Sciences, Section of Biostatistics, University of Genoa, Genova, Italy; 4 Department of Clinical Research, University Hospital Basel, Basel, Switzerland; 5 Service de Neurologie, sclérose en plaques, pathologies de la myéline et neuro-inflammation, Hospices Civils de Lyon, Lyon, France; 6 Université de Lyon, Lyon, France; 7 Observatoire Français de la Sclérose en Plaques, Centre de Recherche en Neurosciences de Lyon, Lyon, France; 8 Eugène Devic EDMUS Foundation against multiple sclerosis, Lyon, France; 9 Department of Translational Biomedicine and Neurosciences - DiBraiN, University of Bari “Aldo Moro”, Bari, Italy; 10 Clinical Neuroscience, Karolinska Institute, Stockholm, Sweden; 11 Independent Healthcare and Real-World Evidence Consultant, Zug, Switzerland; 12 MSBase Foundation, Melbourne, Victoria, Australia; 13 Department of Neurology, The Danish Multiple Sclerosis Registry, Copenhagen University Hospital – Rigshospitalet Glostrup, Glostrup, Denmark; 14 Department of Clinical Medicine, University of Copenhagen, Copenhagen, Denmark; 15 Department of Clinical Epidemiology, Aarhus University Hospital, Aarhus, Denmark; 16 Academic MS Center Zuyd, Department of Neurology, Zuyderland Medical Center, Sittard-Geleen, Netherlands; 17 School for Mental Health and Neuroscience, Department of Neurology, Maastricht University Medical Center, Maastricht, Netherlands; 18 Hôpital Notre Dame, CHUM and Universite de Montreal, Montreal, Quebec, Canada; 19 Hospital Universitario Virgen Macarena, Sevilla, Spain; 20 Hotel-Dieu de Levis, Levis, Quebec, Canada; 21 Department of Neurology and Center of Clinical Neuroscience, Charles University in Prague, 1st Faculty of Medicine and General University Hospital, Prague, Czech Republic; 22 Neurosciences, Hospital Universitari Germans Trias i Pujol, Barcelona, Spain; 23 Clinical Outcomes Research Unit (CORe), Department of Medicine, University of Melbourne, Melbourne, Victoria, Australia; 24 Department of Neurology, Neuroimunology Centre, The Royal Melbourne Hospital, Parkville, Victoria, Australia; 25 Department of Neurosciences, Box Hill Hospital, Box Hill, Victoria, Australia; 26 Eastern Health Clinical School, Monash University, Melbourne, Victoria, Australia; 27 Hunter New England Health, John Hunter Hospital, Newcastle, New South Wales, Australia; 28 Hunter Medical Research Institute, University of Newcastle, Newcastle, New South Wales, Australia; 29 Neurology, Galdakao Hospital, Spain, Spain; 30 Amiri Hospital, Kuwait City, Kuwait; 31 Centre Integre de Sante et de Services Sociaux des Laurentides, Saint-Jerome, Quebec, Canada; 32 South Eastern HSC Trust, Belfast, UK; 33 Department of Neurology, Nancy University Hospital, Université de Lorraine, Nancy, France; 34 Centre Hospitalier Universitaire de Rennes, Rennes, France; 35 Department of Neurology, Centre Hospitalier Universitaire de Toulouse, Toulouse, France; 36 Department of Neurology and Clinical Investigation Center, CHU de Strasbourg and University of Strasbourg, Strasbourg, France; 37 Neurology, AP-HP, Hôpital de la Pitié Salpêtrière, Paris, France; 38 Service de Neurologie, CHU de Bordeaux, Bordeaux, France; 39 Neurocentre Magendie, Université de Bordeaux, Bordeaux, France; 40 CHU de Montpellier, MS Unit, University of Montpellier (MUSE), Montpellier, France; 41 CHU Lille, CRCSEP Lille, Université de Lille, Lille, France; 42 Department of Neurology, Hôpital Saint Vincent de Paul, Lille, France; 43 Department of Neurology, The Alfred Hospital, Melbourne, Victoria, Australia; 44 Department of Neuroscience, School of Translational Medicine, Monash University, Melbourne, Victoria, Australia

**Keywords:** MULTIPLE SCLEROSIS

## Abstract

**Background:**

Effectiveness of disease-modifying treatment (DMT) in people affected by primary progressive multiple sclerosis (PPMS) is limited. Whether specific subgroups may benefit more from DMT in a real-world setting remains unclear. Our aim was to investigate the potential effect of DMT on disability worsening among patients with PPMS stratified by different disability trajectories.

**Methods:**

Within the framework of the Big MS Data network, we merged data from the Observatoire Français de la Sclérose en Plaques, the Swedish and Italian MS registries, and MSBase. We identified patients with PPMS that started DMT or were never treated during the observed period. Subpopulations with comparable baseline characteristics were selected by propensity score matching. Disability outcomes were analysed in time-to-recurrent event analyses, which were repeated in subclasses with different disability trajectories determined by latent class mixed models.

**Results:**

Of the 3243 included patients, we matched 739 treated and 1330 untreated patients with a median follow-up of 3 years after pairwise censoring. No difference in the risk of confirmed disability worsening (CDW) was observed between the groups in the fully matched dataset (HR 1.11, 95% CI 0.97 to 1.23, p=0.127). However, we found a lower risk for CDW among the class of treated patients with an aggressive disability trajectory (n=360, HR 0.68, 95% CI 0.50 to 0.92, p=0.014).

**Conclusions:**

In line with previous studies, our data suggest that DMT does not ameliorate disability worsening in PPMS, in general. However, we observed a beneficial effect of DMT on disability worsening in patients with aggressive predicted disability trajectories.

**WHAT IS ALREADY KNOWN ON THIS TOPIC**

- Effectiveness of disease-modifying treatment (DMT) in primary progressive multiple sclerosis (PPMS) is limited and ocrelizumab is the only drug licensed for the treatment of this condition.

**WHAT THIS STUDY ADDS**

- This study shows that people with PPMS can be classified by their disability trajectories. In those classified as having an aggressively progressing disease, DMT is associated with a lower hazard for disability worsening compared with no treatment.

**HOW THIS STUDY MIGHT AFFECT RESEARCH, PRACTICE OR POLICY**

- This study highlights the importance of identifying patients with PPMS who are likely to accrue disability more rapidly and supports the practice to treat those proactively with DMT.

## Introduction

 Primary progressive multiple sclerosis (PPMS) is characterised by gradual disability accrual from symptom onset, although relapses may occur.^1^ It accounts for 10%–15% of the overall population with MS and carries a worse prognosis compared with the relapsing-remitting disease course.^2 3^ This is also due to the fact that in contrast to relapsing-remitting MS, the response to immunomodulatory treatment is less pronounced, and therefore, a large number of patients remain without disease-modifying treatment (DMT).^4^,^6^ Several disease-modifying drugs have been tested in randomised controlled trials for PPMS, and of these only ocrelizumab demonstrated lower rates of clinical disability worsening, including the risk of requiring a wheelchair, when compared with placebo.^7^,^10^ As randomised clinical trials in progressive MS are challenging to perform and may suffer from limited generalisability due to strict inclusion and exclusion criteria, high-quality observational cohort studies can provide complementary evidence representative of real-world practice.^11 12^ A few large observational studies have examined the effectiveness of DMT in PPMS, with disappointing results in regard to the general PPMS population.^13^,^15^ However, there is mounting evidence that the presence of relapses is a treatable target in PPMS.^13 15^ Still, the majority of patients with PPMS gradually accumulate disability without relapses.

Therefore, our aim was to explore whether there is evidence for a differential effectiveness of DMT on robust disability outcomes in classes of patients with PPMS stratified by distinct disability trajectories.

## Methods

### Participants

Within the framework of the Big MS Data network,^16 17^ data from the Observatoire Français de la Sclérose en Plaques (OFSEP, www.ofsep.org),^18 19^ the Swedish^20^ and Italian MS registries^21^ and the international MSBase registry^22^ were extracted in November 2017. We mapped and unified the variables present in the four datasets, which were then merged into a single dataset (online supplemental methods). Any overlap between the data contained in MSBase and those contained in the national registries was checked and duplicate data were removed. Automated checks on the combined dataset were performed according to the MSBase data quality protocol to ensure data quality and consistency as previously described.^23^

We identified patients with PPMS as defined in the respective cohorts and a minimum available dataset. This consisted of sex, year of birth, year of MS onset, disease course, three Expanded Disability Status Scale (EDSS) assessments recorded after the onset of PPMS, including baseline EDSS (recorded within 6 months from the start of study therapy in treated groups), complete information about MS therapies (treatment name, start and end dates) and relapses (date of relapse onset) and MS centre identifier. For the latent class analyses, an EDSS assessment within 5 years from disease onset was required.

### Outcomes

Outcomes were defined as follows: Disability worsening: EDSS score increase of 1 point (0.5 points if baseline EDSS score ≥6) confirmed at a second visit ≥6 months apart; disability progression: disability worsening in the absence of a relapse (between the visit preceding the disability event and the confirmatory visit); disability reduction: decrease of EDSS score by 1 point (0.5 points if baseline EDSS score ≥6.5) confirmed at a second visit ≥6 months apart. All disability events were assessed using a roving baseline approach. All EDSS scores ≥6 and ≥7 required confirmation over the next ≥6 months. Relapses were defined according to the respective cohorts.

### Statistical analysis

To assign each patient to a disability trajectory class, the following mathematical functions for each class based on EDSS scores and time since disease onset as defined by Signori *et al*^24^ were used:

mild class:


$$EDSS=\begin{array}{l} 1.99+0.032\;\times \;\sqrt{time}\;since\;onset\\ +\;0.12991\times (time\;since\;onset)-0.0026\times time\;since\;onset^{2}; \end{array}$$

moderate class:


$$EDSS=\begin{array}{l} 2.54+0.205\;\times \;\sqrt{time}\;since\;onset\\ +\;0.32\times (time\;since\;onset)-0.07\times time\;since\;onset^{2}; \end{array}$$

severe class:


$$EDSS=\begin{array}{l} 4.24+0.87\;\times \;\sqrt{time}\;since\;onset\\ +\;0.016\times (time\;since\;onset)-0.001\times time\;since\;onset^{2}. \end{array}$$

The first step was to obtain the predicted EDSS at each time point for the three classes. Then, the absolute difference between the observed and the predicted EDSS from each severity class function was calculated for each patient. The prediction closest to the observed EDSS was identified, and the corresponding severity class was defined at each time point. For each patient, the disability class most frequently assigned at all time points represented the final class.

Patients exposed to disease-modifying therapy at any time after the onset of PPMS were allocated to the treated cohort. DMT was defined as interferon beta, glatiramer acetate, teriflunomide, dimethyl fumarate, fingolimod, cladribine, daclizumab, mitoxantrone, natalizumab, alemtuzumab, rituximab, ocrelizumab, ofatumumab and autologous haematopoietic stem cell transplant. Patients were considered to be treated from the recorded treatment start date to the last recorded visit (intention to treat) or recorded treatment end date or the last recorded visit (as-treated). Patients who were not exposed to disease-modifying therapies, any immunosuppressive therapy and who did not take part in a treatment trial were allocated to the untreated cohort.

The study baseline was defined as the first recorded visit with an EDSS assessment for the untreated patients and the start of immunomodulatory treatment for the treated patients. The two groups were matched at baseline, and disability and relapse outcomes were compared between the matched groups. The groups were matched on propensity score, based on a multivariable logistic regression model with treatment allocation as the outcome variable and the demographic and clinical variables available to treating neurologists at baseline as the independent variables.^25^ These comprised sex, age, baseline EDSS, number of relapses in prior 12 months, Multiple Sclerosis Severity Scale (MSSS) at baseline and data source (registry).^26^

Patients were then matched in a variable matching ratio with exact matching on disease duration (in 3-year epochs) by nearest neighbour matching without replacement within a calliper of 0.2 SD of the propensity score. Covariate balance between the matched groups was evaluated using standardised differences (with a difference of <0.2 considered to be acceptable) based on weighted means and medians and percentages (for categorical variables). The common on-treatment follow-up was determined as the shorter of the two available individual follow-up periods for each matched patient pair (pairwise censoring), irrespective of treatment status, to control for attrition bias. All subsequent analyses were designed as paired models adjusted for visit-with-EDSS density (for disability outcomes), with weighting for the variable matching ratio and nested within each centre.

The cumulative hazards of outcome events were evaluated by marginal proportional hazards models for recurrent events with robust estimation of variance, the cluster term indicating the matched pairs.^27^ For confirmed EDSS≥6, EDSS≥7, we applied marginal proportional hazards models for time to single event. Patients with a baseline EDSS≥6 or EDSS≥7, respectively, were excluded from these analyses. Proportionality of hazards was assessed by the Schoenfeld’s global test.

The above analyses were repeated within cohorts stratified into estimated latent disability progression classes (including propensity-score matching within each latent class).

Eight sensitivity analyses were carried out (a) restricting the analysis to patients without relapses prior to baseline, (b) only including patients treated with high-efficacy therapies (mitoxantrone, natalizumab, rituximab, ocrelizumab, alemtuzumab) or untreated, (c) comparing patients treated with high-efficacy therapies with patients on platform injectable drugs (interferon beta, glatiramer acetate), (d) only including patients younger than 45 years at baseline, (e) including only patients for whom a visit with an EDSS assessment was recorded immediately before or after the treatment start (≤60 days) and, EDSS-visit density is ≥1 /year, and not adjusted for visit-with-EDSS density and (f) an ‘as-treated’ analysis, in which pairwise censoring will occur either at change of treatment status or end of follow-up, whichever occurred earlier. By design, our primary analysis was at risk for immortal time bias, that is, treated patients were only observed after the start of DMT, whereas untreated patients contributed to the analysis from their first documented visit. To mitigate potential immortal time bias, we (g) determined the study baseline as the first recorded visit, irrespective of the patient’s treatment status and modelled exposure to DMT as a time-dependent variable. Lastly, we (h) replaced treatment status during the observational period with the proportion of follow-up time spent on disease-modifying agents. The level of evidence for tests of statistical inference was α=0.05.

Data mapping and merging were performed by SS. using R V.4.0, analysis of the latent disability classes by AS using Stata (V.16; StataCorp) and all other analyses by J L using R V.4.0.^28^ R packages used can be found in online supplemental appendix.

## Results

### Main analyses

**Figure 1:**
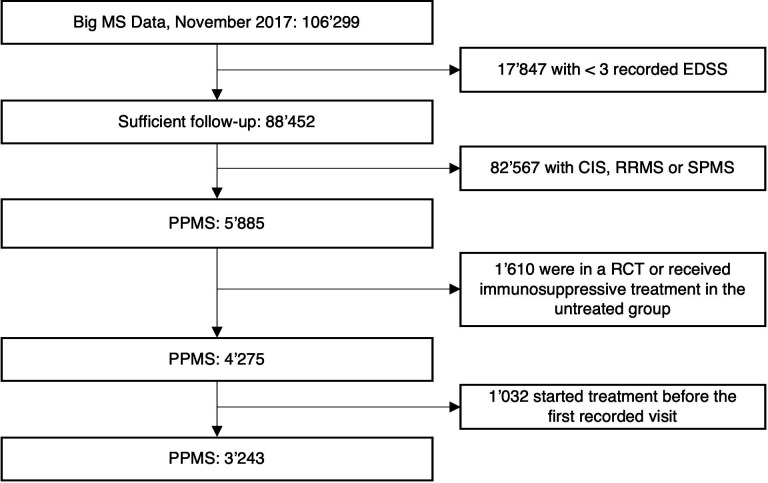
Flow chart of included patients. Included and excluded patients. CIS, clinically isolated syndrome; EDSS, Expanded Disability Status Scale; PPMS, primary progressive multiple sclerosis; RCT, randomised controlled trial; RRMS, relapsing remitting multiple sclerosis; SPMS, secondary progressive multiple sclerosis.

We included 3243 patients with PPMS in the primary analysis (figure 1). Patient characteristics differed markedly between the treated (n=1181) and untreated groups (n=2067, table 1). Younger age, shorter disease duration and lower EDSS scores, but faster disability accumulation as measured by the MSSS, and higher relapse rates were associated with a higher propensity of being treated (online supplemental table). Of note, the majority of patients (n=657, 56%) were treated with platform injectable therapies, relatively few (n=123, 12%) were on B-cell depleting drugs, and of these only a minority were treated with ocrelizumab, which is the only approved drug treatment for PPMS (n=23, 3%). The propensity score-based matching procedure retained 739 treated and 1330 untreated patients and improved the overall balance between the groups by 97% (online supplemental figure). The median follow-up time after pairwise censoring was approximately 3 years (IQR 1.8–4.9 years).

**Table 1:** Patient characteristics before and after matching

	Unmatched			Matched		
	Untreated	Treated	SMD	Untreated	Treated	SMD
n	2062	1181		1330	739	
Age, mean, years (SD)	53.67 (10.1)	44.87 (10.49)	0.86	50.3 (9.5)	49.6 (9.2)	0.19
Gender female, n (%)	1185 (57%)	601 (51%)	0.13	765 (57%)	382 (52%)	0.07
Disease duration, mean, years, SD	9.12 (8.35)	7.16 (6.12)	0.27	6.5 (5.9)	6.4 (5.6)	0.03
EDSS, median (quartiles)	4.5 (3.0, 6.0)	4.0 (3.0, 5.0)	0.33	4.0 (3.0, 6.0)	4.0 (3.0, 5.5)	0.03
MSSS, median (quartiles)	7.3 (5.4, 8.6)	7.9 (5.9, 8.8)	0.28	7.5 (5.8, 8.7)	7.9 (6.0, 9.0)	0.13
Relapses in the prior year, mean (SD)	0.03 (0.19)	0.21 (0.52)	0.46	0.07 (0.30)	0.05 (0.23)	0.10
Treatment at baseline						
Dimethyl fumarate, n (%)		9 (<1%)			4 (<1%)	
Fingolimod, n (%)		58 (5%)			42 (5%)	
Glatiramer acetate, n (%)		145 (12%)			115 (15%)	
Interferon beta, n (%)		512 (43%)			345 (47%)	
Mitoxantrone, n (%)		310 (26%)			172 (22%)	
Natalizumab, n (%)		16 (1%)			10 (1%)	
Ocrelizumab, n (%)		29 (3%)			21 (3%)	
Rituximab, n (%)		94 (8%)			93 (8%)	
Teriflunomide, n (%)		8 (<1%)			6 (<1%)	
Dataset			0.58			0.16
OFSEP, n (%)	770 (37%)	743 (63%)		613 (46%)	414 (53%)	
Swedish MS registry, n (%)	487 (24%)	151 (13%)		262 (20%)	121 (16%)	
Italian MS registry, n (%)	289 (14%)	161 (14%)		201 (15%)	118 (15%)	
MSBase, n (%)	516 (25%)	126 (11%)		254 (19%)	125 (16%)	
Follow-up, years median (quartiles)	6.8 (3.9, 10.9)	5.8 (3.0, 10.3)		2.8 (2.1, 4.0)*	2.9 (1.8, 4.9)*	

* After pairwise censoring.

EDSS, Expanded Disability Status Scale; MSSS, Multiple Sclerosis Severity Scale; OFSEP, Observatoire Français de la Sclérose en Plaques; SMD, standardised mean difference.

**Figure 2:**
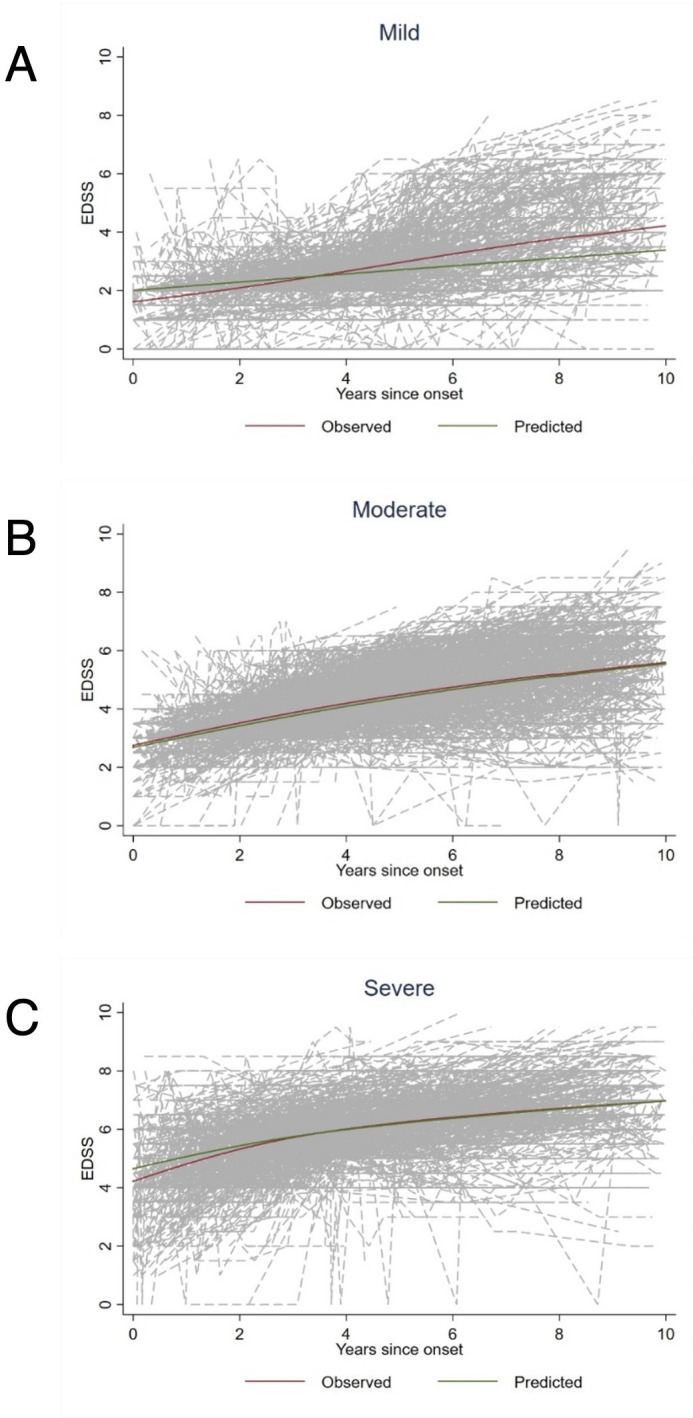
Disability trajectories. Disability trajectories determined by latent class analysis. Individual patients’ trajectories (grey), observed trajectories (red) and predicted trajectories (green). (**A**) Mild disability trajectory, n=366; (**B**) Moderate disability trajectory, n=681; (**C**) Severe disability trajectory, n=571. EDSS, Expanded Disability Status Scale.

For the latent class analysis, we could include 1618 patients. Of these, 366 were categorised in the mild class, 681 were classified as moderate and 571 were classified as severe (figure 2). Patient characteristics of the three different classes are presented in table 2: Age, sex and disease duration were distributed relatively similarly among the three classes. However, EDSS and MSSS increased markedly from the moderate to the severe class, and a lower proportion of patients in the mild class were exposed to immunomodulatory treatments. The greatest difference could be found for mitoxantrone, which was only used in 15% of patients in the mild group, but in 34% of patients in the severe class.

**Table 2:** Baseline characteristics of patients with different disability trajectories

	Mild	Moderate	Severe
n	366	681	571
Age, mean, years (SD)	46.3 (10.8)	46.4 (10.7)	48.1 (10.9)
Sex, female, n (%)	186 (51)	354 (52)	322 (56)
Disease duration, mean, years, SD	3.5 (3.7)	3.4 (2.6)	2.8 (2.3)
EDSS, median (quartiles)	2.5 (2.0, 3.0)	4.0 (3.5, 4.0)	6.0 (4.5, 6.0)
MSSS, median (quartiles)	5.9 (4.8, 6.8)	8.0 (7.3, 8.6)	9.2 (8.8,10.0)
Relapses in the prior year, mean (SD)	0.10 (0.34)	0.15 (0.45)	0.14 (0.41)
Treated, n (%)	147 (40)	347 (51)	286 (50)
Dimethyl fumarate, n (%)	3 (2)	3 (<1)	1 (<1)
Fingolimod, n (%)	6 (4)	17 (5)	16 (6)
Glatiramer acetate, n (%)	18 (12)	52 (15)	28 (10)
Interferon beta, n (%)	80 (22)	152 (22)	103 (18)
Mitoxantrone, n (%)	22 (15)	85 (24)	97 (34)
Natalizumab, n (%)	3 (2)	2 (<1)	5 (2)
Ocrelizumab, n (%)	0 (0)	14 (4)	9 (3)
Rituximab, n (%)	13 (9)	21 (6)	25 (9)
Teriflunomide, n (%)	2 (1)	1 (<1)	2 (<1)

EDSS, Expanded Disability Status Scale; MSSS, Multiple Sclerosis Severity Scale.

**Figure 3:**
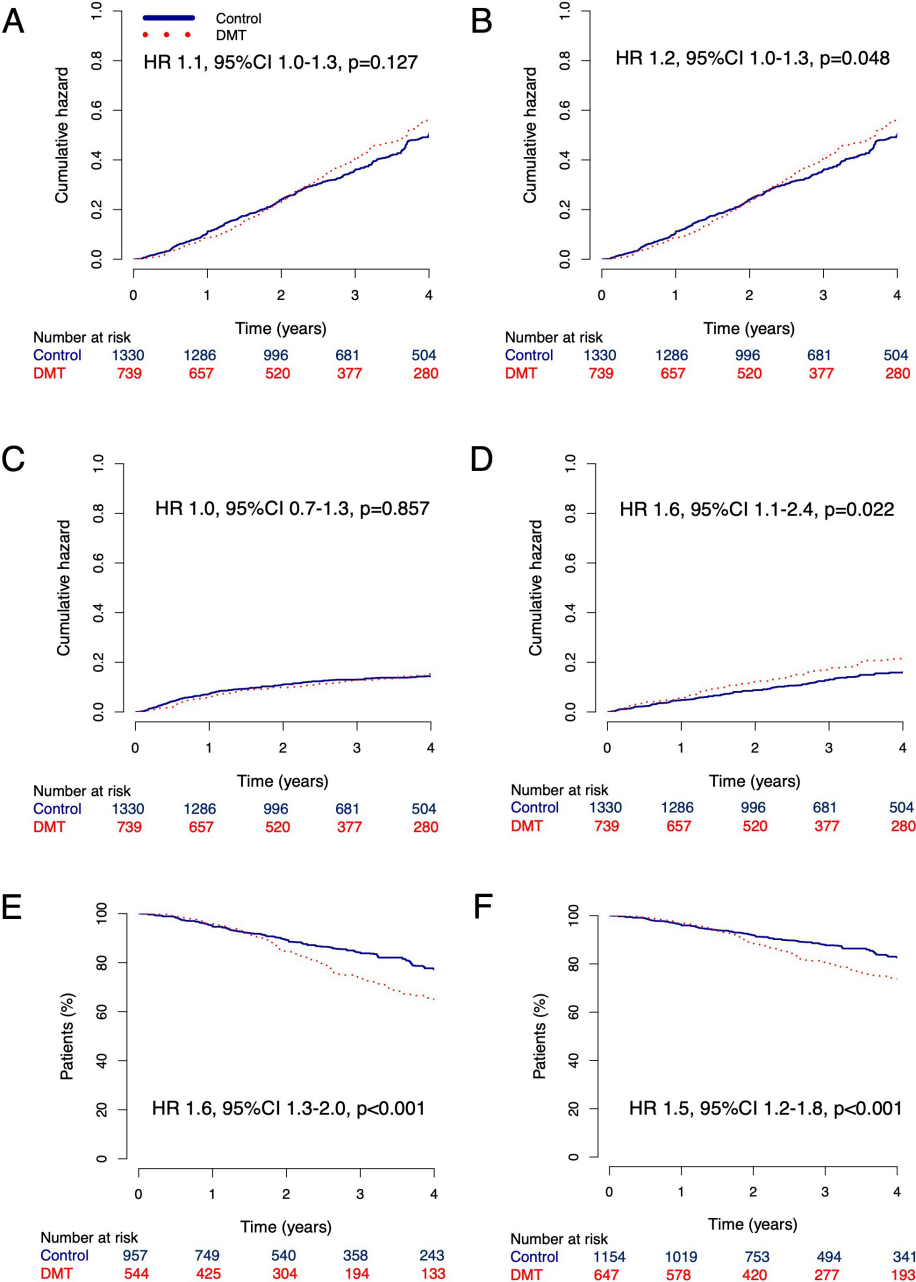
Clinical outcomes of the primary analysis. Clinical outcomes in matched patients. (**A**) Cumulative hazard for disability worsening, (**B**) cumulative hazard for disability progression, (**C**) cumulative hazard for disability improvement, (**D**) cumulative hazard for relapses, (**E**) percentage of patients who do not reach an EDSS≥6, (**F**) percentage of patients who do not reach an EDSS≥7. DMT, disease-modifying treatment; EDSS, Expanded Disability Status Scale.

In the matched analysis of the primary dataset, we found that treated and untreated patients had a similar risk of experiencing overall disability worsening (HR 1.11, 95% CI 0.97 to 1.28 p=0.127) and disability improvement (HR 0.97, 95% CI 0.73 to 1.30, p=0.857). However, we observed a higher cumulative risk for disability progression independent of relapses (HR 1.15, 95% CI 1.00 to 1.32 p=0.048), experiencing relapses (1.59, 95% CI 1.07 to 2.36, p=0.022) and faster progression to EDSS 6 (HR 1.56, 95% CI 1.25 to 1.96, p<0.001) and EDSS 7 (HR 1.45, 95% CI 1.18 to 1.78, p<0.001) in treated compared with untreated patients (figure 3).

When we performed the prespecified subclass analysis in patients with a mild disability trajectory, no between-group differences were observed between the treated and the untreated group (table 3). In the moderate class, we found a higher chance of disability improvement among the treated patients. In contrast, we found a lower cumulative hazard for disability worsening and disability progression in patients with an aggressive disability trajectory who were treated with a disease-modifying drug, as well as a higher chance of disability improvement.

**Table 3:** Outcomes in subclasses with different disability trajectories

Mild: matched 70 treated, 150 untreated	
Disability worsening	HR 1.91, 95% CI 0.92 to 4.00, p=0.086
Disability progression	HR 1.51, 95% CI 1.00 to 2.30, p=0.051
Disability improvement	HR 0.84, 95% CI 0.46 to 1.55, p=0.583
Relapses	HR 1.05 95% CI 0.46 to 2.44, p=0.902
EDSS 6	HR 2.20, 95% CI 0.87 to 5.53, p=0.098
EDSS 7	HR 2.19, 95% CI 0.87 to 5.53, p=0.098
Moderate: matched 168 treated, 282 untreated	
Disability worsening	HR 1.22, 95% CI 0.85 to 1.75, p=0.276
Disability progression	HR 1.14, 95% CI 0.85 to 1.54, p=0.396
Disability improvement	HR 2.36, 95% CI 1.26 to 4.43, p=0.007
Relapses	HR 1.28, 95% CI 0.71 to 2.31, p=0.394
EDSS 6	HR 1.27, 95% CI 0.84 to 1.91, p=0.264
EDSS 7	HR 1.26, 95% CI 0.84 to 1.90, p=0.270
Severe: matched 131 treated, 229 untreated	
Disability worsening	HR 0.68, 95% CI 0.50 to 0.92, p=0.014
Disability progression	HR 0.69, 95% CI 0.50 to 0.96, p=0.030
Disability improvement	HR 2.11, 95% CI 1.05 to 4.23, p=0.035
Relapses	HR 1.50, 95% CI 0.64 to 3.49, p=0.342
EDSS 6	HR 0.92, 95% CI 0.54 to 1.57, p=0.766
EDSS 7	HR 0.95, 95% CI 0.60 to 1.48, p=0.819

EDSS, Expanded Disability Status Scale.

### Sensitivity analyses

The sensitivity analysis of patients without relapse activity before inclusion confirmed the results of the primary analysis (table 4). Comparing patients on high-efficacy treatments with untreated patients, we found no difference in disability outcomes, but a lower risk for relapses. In patients below the age of 45 years, treatment was associated with a shorter time to reaching the disability milestones EDSS 6 or 7. When we defined the study baseline as the first recorded visit in both groups and modelled exposure to DMT as a time-dependent variable to mitigate potential immortal time bias, the results of the primary analysis were confirmed. Replacing treatment status at inclusion with the proportion of follow-up time spent on treatment only showed a higher risk for relapses in the treated group. In summary, most sensitivity analyses confirmed the results of the primary analysis. However, the ‘as-treated’ approach showed a lower risk for disability worsening and progression among patients on immunomodulatory treatments, but no difference in the time to reach an EDSS≥6 or 7.

**Table 4:** Sensitivity analyses

No relapses prior to baseline: matched 667 treated, 1248 untreated	
Disability worsening	HR 1.46, 95% CI 1.18 to 1.81, p<0.001
Disability progression	HR 1.17, 95% CI 1.01 to 1.35, p=0.011
Disability improvement	HR 1.47, 95% CI 1.01 to 1.99, p=0.035
Relapses	HR 1.37, 95% CI 0.92 to 2.05, p=0.123
EDSS 6	HR 1.52, 95% CI 1.20 to 1.91, p<0.001
EDSS 7	HR 1.42, 95% CI 1.14 to 1.76, p<0.001
High-efficacy treatments vs untreated: matched 297 treated, 693 untreated	
Disability worsening	HR 1.10, 95% CI 0.78 to 1.55, p=0.590
Disability progression	HR 1.10, 95% CI 0.88 to 1.38, p=0.411
Disability improvement	HR 1.05, 95% CI 0.66 to 1.65, p=0.849
Relapses	HR 0.47, 95% CI 0.24 to 0.92, p=0.029
EDSS 6	HR 1.37, 95% CI 0.94 to 1.97, p=0.094
EDSS 7	HR 1.17, 95% CI 0.80 to 1.58, p=0.490
High-efficacy treatments vs platform: matched 322 high-efficacy, 523 platform	
Disability worsening	HR 1.11, 95% CI 0.82 to 1.50, p=0.485
Disability progression	HR 1.04, 95% CI 0.81 to 1.34, p=0.763
Disability improvement	HR 1.41, 95% CI 0.94 to 2.14, p=0.094
Relapses	HR 0.67, 95% CI 0.37 to 1.19, p=0.170
EDSS 6	HR 1.05, 95% CI 0.74 to 1.48, p=0.776
EDSS 7	HR 1.08, 95% CI 0.79 to 1.47, p=0.620
Age <45: matched 252 treated, 306 untreated	
Disability worsening	HR 1.21, 95% CI 0.86 to 1.70, p=0.296
Disability progression	HR 1.10, 95% CI 0.87 to 1.39, p=0.419
Disability improvement	HR 1.31, 95% CI 0.81 to 2.12, p=0.262
Relapses	HR 1.43, 95% CI 0.79 to 2.59, p=0.236
EDSS 6	HR 1.50, 95% CI 1.05 to 2.14, p=0.025
EDSS 7	HR 1.40, 95% CI 1.00 to 1.94, p=0.047
EDSS assessment ≤60 days before or after treatment start, EDSS-visit density ≥1/year, not adjusted for visit-with-EDSS density. Matched 729 treated, 1330 untreated	
Disability worsening	HR 1.17, 95% CI 1.02 to 1.32, p=0.023
Disability progression	HR 1.16, 95% CI 1.01 to 1.33, p=0.039
Disability improvement	HR 0.98, 95% CI 0.73 to 1.31, p=0.879
Relapses	HR 1.58, 95% CI 1.07 to 2.36, p=0.023
EDSS 6	HR 1.57, 95% CI 1.26 to 1.95, p<0.001
EDSS 7	HR 1.45, 95% CI 1.18 to 1.78, p<0.001
As-treated analysis: matched 739 treated, 1330 untreated	
Disability worsening	HR 0.85, 95% CI 0.73 to 0.97, p=0.021
Disability progression	HR 0.84, 95% CI 0.72 to 0.98, p=0.032
Disability improvement	HR 1.13, 95% CI 0.82 to 1.55, p=0.462
Relapses	HR 1.32, 95% CI 0.87 to 1.99, p=0.192
EDSS 6	HR 1.14, 95% CI 0.89 to 1.45, p=0.302
EDSS 7	HR 1.01, 95% CI 0.80 to 1.27, p=0.926
First recorded visit as study baseline for treated and untreated patients. Exposure to DMT modelled as time-dependent variable: matched 898 treated, 1461 untreated	
Disability worsening	HR 1.38, 95% CI 1.23 to 1.55, p<0.001
Disability progression	HR 1.35, 95% CI 1.19 to 1.53, p<0.001
Disability improvement	HR 1.83, 95% CI 1.40 to 2.41, p<0.001
Relapses	HR 1.58, 95% CI 1.14 to 2.19, p=0.006
EDSS 6	HR 2.59, 95% CI 2.11 to 3.18, p<0.001
EDSS 7	HR 2.45, 95% CI 2.02 to 2.97, p<0.001
Replacing treatment status at inclusion with the proportion of follow-up time spent on DMT: matched 739 treated, 1330 untreated	
Disability worsening	HR 1.11, 95% CI 0.91 to 1.36, p=0.297
Disability progression	HR 1.12, 95% CI 0.91 to 1.38, p=0.050
Disability improvement	HR 1.03, 95% CI 0.67 to 1.58, p=0.904
Relapses	HR 1.74, 95% CI 1.08 to 2.78, p=0.022
EDSS 6	HR 1.28, 95% CI 0.95 to 1.72, p=0.102
EDSS 7	HR 1.25, 95% CI 0.93 to 1.66, p=0.135

DMT, disease-modifying treatment; EDSS, Expanded Disability Status Scale.

## Discussion

In this large combined retrospective analysis of prospectively acquired data from the Big MS Data network, we found that in the overall population of patients with PPMS, exposure to immunomodulatory treatment was associated with a higher risk for disability progression and for reaching important disability milestones in the medium term. In contrast, in the prespecified subclass with aggressive disability trajectories, treated patients had a lower risk for disability worsening and progression. A series of sensitivity analyses generally confirmed the results of the primary analysis. However, in the ‘as-treated’ analysis, DMT was associated with a reduced risk for disability worsening and progression, suggesting that observation in the matched cohort restricted to the treated epochs, showed a small benefit of DMT, but only as long as patients were on therapy.

The observation that DMT is associated with worse disability outcomes in patients suffering from PPMS has also been made in a recent analysis from the Italian MS Registry by Portaccio *et al*.^15^ In both their and our studies, this finding is most likely caused by residual treatment indication bias that could not be completely overcome by the propensity score matching procedure.

However, and more importantly, Portaccio *et al* showed that DMT was associated with a lower risk of reaching EDSS 7, that is, the essential loss of ambulatory function, in patients with PPMS that presented inflammatory activity. In their matched cohort, a rather large proportion of patients had clinical relapses in the 2 years before study inclusion (16–18%) and during the observational period (36%). When limiting the analysis to those patients who did not exhibit any relapse activity in the year before baseline, an association between superimposed relapses over the follow-up period and treatment effectiveness remained, but did not reach the level of statistical evidence. So, the authors concluded that inflammatory activity was a modifiable risk factor of long-term disability in PPMS, and the presence of relapses could be used to guide treatment decisions in these patients.

The finding that patients with superimposed relapses seem to benefit from immunomodulatory treatment is also in line with a previous study from the MSBase registry, which showed that the proportion of follow-up time spent on disease-modifying therapy significantly reduced the hazard of confirmed disability progression in PPMS patients with, but not in those without relapses.^13^

In our analysis, the relapse-rates in the year before inclusion were considerably lower than in the Italian study, which prevented us from analysing this group separately due to limited analytical power. However, our sensitivity analysis of patients without relapse activity prior to baseline is in line with the results of Portaccio *et al,* showing worse disability outcomes for patients on DMT. Given our study design, we were not able to stratify the analysis for the presence of relapses during the prospective follow-up time, as this would have introduced conditioning on future events. Therefore, we chose two separate disability outcomes, that is, overall disability worsening and disability progression, which occurred exclusively in the absence of relapse activity. Still, these two outcomes showed very similar results throughout our suite of analyses.

However, the focus of our analysis was to explore whether patients with different disability trajectories might respond differently to immunomodulatory treatment. To this end, we used the approach previously developed by Signori *et al* and performed a latent class growth analysis on the longitudinal disability data.^24^ This method assigns patients to classes depending on the patients’ disability trajectories taking into account disability level, that is, absolute EDSS values and change over time in relation to disease onset.

Similarly, as in the original study, which was based on data from the MSBase registry only, we could allocate patients to three distinct classes with mild, moderate or severe disability trajectories.

In the severely affected patients, the disease had caused more disability in shorter time already at study baseline and continued to do so during the observational period. Still, DMT exposure was associated with a lower risk of disability worsening and progression in these patients. This is even more striking, considering the results of the primary analysis of the entire matched cohort, in which patients treated with DMT fared relatively worse than the untreated ones. One potential reason for this finding could be the fact that highly effective treatment was used more frequently in patients with severe disability trajectories. In our cohort, this was mostly due to a larger proportion of patients treated with mitoxantrone, whereas the use of B-cell depleting drugs was relatively similarly distributed among the three groups. Of note, relapses in the year before inclusion occurred rarely and at a similar rate in all three groups (0.10–0.14 relapses per patient-year).

We performed a series of sensitivity analyses to investigate the robustness of the results from the primary analysis in relation to variations of our inclusion and exclusion criteria and statistical methods. Generally, these confirmed the results of the primary analysis of the entire cohort. High efficacy treatment was associated with a lower cumulative risk for relapses compared with no treatment and platform DMT but not with improved disability outcomes. When restricting the study population to patients younger than 45 years at baseline, we did not find any relevant differences compared with the primary analysis. This is in contrast to previous studies, which have shown a better treatment response also in regard to reducing disability worsening in younger patients^29 30^ but might be due to limited analytical power and the relatively low relapse activity observed in our cohort.

One notable exception to the general trend was the ‘as-treated’ analysis that evaluated immediate outcomes during treatment exposure, not influenced by potential confounding during the untreated epochs. In this analysis, we found a lower cumulative hazard for disability worsening and progression in the treated patients, but the hazard of reaching the disability milestones EDSS 6 and 7 was similar in both groups. By removing the effect of untreated epochs, these findings suggest that even in the entire matched cohort, patients on therapy had a lower risk for disability accrual, but only while being treated. These results, therefore, provide an argument for sustained use of DMT in PPMS, if it is to be beneficial.

Our study has several limitations. The most important one is the observational study design and the lack of randomisation. To mitigate the known treatment indication bias, we employed propensity score matching. However, propensity score matching does not eliminate unknown confounders and, as in our case, can still result in residual imbalance if a strong treatment indication bias is present. However, our study used strict matching criteria, including exact matching on disease duration and two disability metrics. Pairwise censoring was applied to control for attrition bias. We also adjusted for reporting bias by considering the frequency of follow-up visits. To mitigate potential immortal time bias, we added a sensitivity analysis, for which the study baseline was defined as the first recorded visit for both treated and untreaded patients and exposure to DMT was modelled as a time-dependent variable, which confirmed the results of the primary analysis.

Another limitation is the relatively limited follow-up after pairwise censoring. With a median follow-up of approximately 3 years, we were unable to assess long-term outcomes and might miss a delayed effect of DMT.

Combining observational datasets from four different sources will have increased the heterogeneity of the data. To mitigate this effect, we applied a strict protocol for data cleaning and harmonisation and generated a large dataset of patients with PPMS with substantial follow-up and broad generalisability.

As MRI information was unavailable in the combined dataset, we could not match patients on MRI activity, analyse potential subgroup effects in patients with radiologically active disease or study treatment effects on MRI outcomes.

Our primary disability outcomes also reflect the inherent limitations of the EDSS. The EDSS relies heavily on lower limb function and its sensitivity to cognitive changes and upper limb function in more advanced MS is relatively low.^31^ In addition, the EDSS is burdened with relatively low intrarater and inter-rater reliability, especially at the lower end of the scale.^32 33^ However, the use of EDSS-based metrics enabled us to analyse robust disability outcomes and relevant disability milestones.

In conclusion, our study, using a large observational dataset from the Big MS Data network confirmed previous work suggesting that DMT in general is not associated with better disability outcomes in people with PPMS. However, it highlights the importance of viewing PPMS as a heterogeneous group of patients with varying degrees of focal inflammation. Consequently, treatment strategies should not be based on the presenting symptoms only but should include the identification of PPMS patients who are likely to accumulate disability more rapidly to treat those proactively with DMT.

## Supplementary material

10.1136/jnnp-2024-334700 online supplemental file 1

10.1136/jnnp-2024-334700 online supplemental file 2

## Data Availability

Data may be obtained from a third party and are not publicly available.

## References

[1] Miller DH, Leary SM (2007). Primary-progressive multiple sclerosis. Lancet Neurol.

[2] Amezcua L (2022). Progressive Multiple Sclerosis. Continuum (Mount Lawley).

[3] Koch M, Kingwell E, Rieckmann P (2009). The natural history of primary progressive multiple sclerosis. Neurology (ECronicon).

[4] Stuve O, Paul F (2013). Progressive multiple sclerosis: desperately seeking remedy. Lancet Neurol.

[5] Diouf I, Malpas CB, Sharmin S (2023). Variability of the response to immunotherapy among subgroups of patients with multiple sclerosis. Eur J Neurol.

[6] Watson C, Thirumalai D, Barlev A (2023). Treatment Patterns and Unmet Need for Patients with Progressive Multiple Sclerosis in the United States: Survey Results from 2016 to 2021. Neurol Ther.

[7] Montalban X, Hauser SL, Kappos L (2017). Ocrelizumab versus Placebo in Primary Progressive Multiple Sclerosis. N Engl J Med.

[8] Lublin F, Miller DH, Freedman MS (2016). Oral fingolimod in primary progressive multiple sclerosis (INFORMS): a phase 3, randomised, double-blind, placebo-controlled trial. Lancet.

[9] Wolinsky JS, Narayana PA, O’Connor P (2007). Glatiramer acetate in primary progressive multiple sclerosis: results of a multinational, multicenter, double-blind, placebo-controlled trial. Ann Neurol.

[10] Butzkueven H, Spelman T, Horakova D (2022). Risk of requiring a wheelchair in primary progressive multiple sclerosis: Data from the ORATORIO trial and the MSBase registry. Eur J Neurol.

[11] Koch MW, Cutter G, Stys PK (2013). Treatment trials in progressive MS--current challenges and future directions. Nat Rev Neurol.

[12] Trojano M, Tintore M, Montalban X (2017). Treatment decisions in multiple sclerosis - insights from real-world observational studies. Nat Rev Neurol.

[13] Hughes J, Jokubaitis V, Lugaresi A (2018). Association of Inflammation and Disability Accrual in Patients With Progressive-Onset Multiple Sclerosis. JAMA Neurol.

[14] Lorscheider J, Kuhle J, Izquierdo G (2019). Anti-inflammatory disease-modifying treatment and disability progression in primary progressive multiple sclerosis: a cohort study. Eur J Neurol.

[15] Portaccio E, Fonderico M, Iaffaldano P (2022). Disease-Modifying Treatments and Time to Loss of Ambulatory Function in Patients With Primary Progressive Multiple Sclerosis. JAMA Neurol.

[16] Hillert J, Magyari M, Soelberg Sørensen P (2021). Treatment Switching and Discontinuation Over 20 Years in the Big Multiple Sclerosis Data Network. Front Neurol.

[17] Iaffaldano P, Lucisano G, Butzkueven H (2021). Early treatment delays long-term disability accrual in RRMS: Results from the BMSD network. Mult Scler.

[18] Vukusic S, Casey R, Rollot F (2020). Observatoire Français de la Sclérose en Plaques (OFSEP): A unique multimodal nationwide MS registry in France. Mult Scler.

[19] Confavreux C, Compston DA, Hommes OR (1992). EDMUS, a European database for multiple sclerosis. *J Neurol Neurosurg Psychiatry*.

[20] Hillert J, Stawiarz L (2015). The Swedish MS registry – clinical support tool and scientific resource. Acta Neurol Scand.

[21] Trojano M, Bergamaschi R, Amato MP (2019). The Italian multiple sclerosis register. Neurol Sci.

[22] Butzkueven H, Chapman J, Cristiano E (2006). MSBase: an international, online registry and platform for collaborative outcomes research in multiple sclerosis. Mult Scler.

[23] Kalincik T, Kuhle J, Pucci E (2017). Data quality evaluation for observational multiple sclerosis registries. Mult Scler.

[24] Signori A, Izquierdo G, Lugaresi A (2018). Long-term disability trajectories in primary progressive MS patients: A latent class growth analysis. Mult Scler.

[25] Rosenbaum PR, Rubin DB (1984). Reducing Bias in Observational Studies Using Subclassification on the Propensity Score. J Am Stat Assoc.

[26] (2005). Multiple Sclerosis Severity Score: using disability and disease duration to rate disease severity. Neurol (ECronicon).

[27] Andersen PK, Gill RD (1982). Cox’s Regression Model for Counting Processes: A Large Sample Study. Ann Statist.

[28] Team RDC (2011). R: a language and environment for statistical computing.

[29] Hawker K, O’Connor P, Freedman MS (2009). Rituximab in patients with primary progressive multiple sclerosis: results of a randomized double-blind placebo-controlled multicenter trial. Ann Neurol.

[30] Amato MP, Fonderico M, Portaccio E (2020). Disease-modifying drugs can reduce disability progression in relapsing multiple sclerosis. Brain (Bacau).

[31] Amato MP, Portaccio E (2007). Clinical outcome measures in multiple sclerosis. J Neurol Sci.

[32] Amato MP, Fratiglioni L, Groppi C (1988). Interrater reliability in assessing functional systems and disability on the Kurtzke scale in multiple sclerosis. Arch Neurol.

[33] Goodkin DE, Cookfair D, Wende K (1992). Inter- and intrarater scoring agreement using grades 1.0 to 3.5 of the Kurtzke Expanded Disability Status Scale (EDSS). Multiple Sclerosis Collaborative Research Group. Neurology (ECronicon).

