# Supplementary material for: Disease-modifying treatment and disability progression in subclasses of patients with primary progressive MS: results from the Big MS Data Network
Source: J Neurol Neurosurg Psychiatry. 2024 Dec 6;96(6):e334700. doi: 10.1136/jnnp-2024-334700 (PMC12171517; doi:10.1136/jnnp-2024-334700)
Supplement: online supplemental file 1 [file jnnp-96-6-s001.pdf]

Supplement - Data preparation

1. Data mapping and merging

Data were extracted from the following registries in November 2017:

- Observatoire Français de la Sclérose en Plaques (OFSEP)
- The Italian MS Registry
- The Danish MS Registry
- The Swedish MS Registry
- MSBase

An „R" package was created to contain functions for loading, saving and formatting data. The package also internally contains a codebook to recode treatment entries and a drug list to assign treatment effect durations. These data are loaded when the package is installed.

The annotated R code used for merging is available on GitHub.

The data files were shared in 5 different folders, 1 for each cohort. Within each folder, data is contained in different files, e.g. Patient, Visits, Relapses, Treatment, Lab and MRI. The formats of the files vary between cohorts e.g. xlsx, csv, txt or sas7bdat. Some files had encoding issues. For example encoding "latin1" was used to load data from Sweden and France.

Data were organized in 4 combined tables:

- Patients
- Visits
- Relapses
- Treatments

1.1 "Patients" table

The following variables were selected from the "Patients" tables of each cohort where available:

- Patient ID
- Date of birth
- Gender
- Country
- Date of first symptoms or first relapse
- Death of death
- Start of progression
- MS diagnosis date
- MS course
- Mc Donald classification

A new, unique Patient ID variable containing the data source was created. 1. The date columns were formatted using the function bigMS::format.date.multiple.col(). Empty strings were replaced with NAs and factors/numerics/integers were converted to characters. Duplicates were removed. For MSBase cohort, the variables gender, birth date, country, first symptoms date and diagnosis date were used to

detect duplicates. For the other cohorts, only the patient id was used. A new variable called "CURRENT_MSCOURSE" was created to display the most recent MS course of the patient. Finally, logical checks were performed on MS course information.

1.2 "Visits" table

Variables selected:

- Patient ID
- Date of visit
- EDSS
- Functional system scores 1-7 and Ambulation Score

The date columns were formatted using the function bigMS::format.date.multiple.col(). The Kurtzke Functional System (KFS) Scores were renamed and formatted as integers. Factors/numerics/integers were converted to characters.

1.3 "Relapses" table

Variables selected:

- Patient ID
- Date of onset
- Symptoms(pyramidal, sensory, cerebellar, visual, brainstem, bowel/bladder, cognitive) PYRAMIDAL_TRACT
- Corticosteroids given

The date columns were formatted using the function bigMS::format.date.multiple.col(). Factors/numerics/integers were converted to characters.

1.4 "Treatments" table

Variables selected:

1. Patient ID
2. Treatment name
3. Treatment start date
4. Treatment end date

The date columns were formatted using the function bigMS::format.date.multiple.col(). Empty strings were replaced with NAs and factors/numerics/integers were converted to characters. Treatments were converted to lower case in order to be matched with the treatment codebook. Where applicable, Treatment columns were converted to UTF8 encoding.

New variables created:

- Compound name: Generic names for all DMT, immunosuppressive treatments

- Treatment groups
  - DMT
  - Immunosuppressive treatment
  - Hematopoietic stem cell transplantation
  - Randomized controlled trial

2. Data quality

2.1 Data quality procedure

- Duplicate patient records were removed.
- MS onset dates after the data extract dates were removed.
- The dates of MS onset and the first recorded MS course were aligned.
- Patients with the age at onset outside the 0-100 range were excluded.
- A logical sequence of the MS courses (e.g. clinically isolated syndrome, relapsing remitting MS, secondary progressive MS) was assured.
- Entries with the initiation of progressive MS prior to its clinical onset of MS were excluded.
- Visits with missing visit date or the recorded date before the clinical MS onset or after the date of MSBase data extract were removed.
- EDSS scores outside the range of possible EDSS values were removed.
- Duplicate visits were merged.
- MS relapses with missing visit date or the recorded date after the date of data extract were removed.
- Duplicate MS relapses were merged.
- Relapses occurring within 30 days of each other were merged.
- Visits preceded by relapses were identified and time from the last relapse was calculated for each visit.
- Therapies with erroneous date entries were removed (e.g. commencement date > termination date, commencement after the data extract date, commencement of disease modifying therapy before the year 1980).
- Duplicate treatment entries were removed.
- Where multiple disease modifying therapies are recorded simultaneously, treatment end date of the previous therapy were imputed as the commencement date of the following therapy.
- Consecutive entries for certain disease modifying therapies were merged into a continuous treatment entry, given that the gap between the entries does not exceed 190 days for mitoxantrone, 365 days for cladribine, 280 for ocrelizumab and rituximab, and 90 days for other disease modifying therapies.
- The default duration of treatment effect was recorded as 190 days (mitoxantrone), 5 years (alemtuzumab), 280 days (ocrelizumab and rituximab) or 365 days (cladribine) from treatment commencement.

2.2 Data quality log

- All patients: 115368
- Present and plausible date of birth: 115357
- Present and plausible MS onset date: 113363
- Plausible sequence of the MS course dates: 113349
- Present and plausible dates for onset of progression: 113295
- Patient records excluded: 2073
- Visits excluded/merged: 142381
- Relapses excluded/merged: 8518
- Treatments excluded/merged: 39277
