# Supplementary material for: Disease-modifying treatment and disability progression in subclasses of patients with primary progressive MS: results from the Big MS Data Network
Source: J Neurol Neurosurg Psychiatry. 2024 Dec 6;96(6):e334700. doi: 10.1136/jnnp-2024-334700 (PMC12171517; doi:10.1136/jnnp-2024-334700)
Supplement: online supplemental file 2 [file jnnp-96-6-s002.pdf]

Supplementary files

Supplementary table: Outcomes of the logistic regression model for propensity score matching

**Independent variable**	**Coefficient**	**Std. error**	**p-value**
Male patient sex	0.156	0.086	0.069
Age at baseline	-0.072	0.004	<0.001
Disease duration at baseline	-0.306	0.029	<0.001
EDSS at baseline	-0.599	0.047	<0.001
MSSS at baseline	0.505	0.038	<0.001
Relapses in the year before baseline	1.128	0.151	<0.001
Italian MS registry*	-0.244	0.128	0.058
MSBase*	-1.095	0.129	<0.001
Swedish MS registry*	-0.518	0.122	<0.001

Logistic regression model for the propensity of treatment vs. no treatment (reference).
EDSS = Expanded Disability Status Scale, MSSS = Multiple Sclerosis Severity Scale
*OFSEP used as reference value.

Supplementary figure

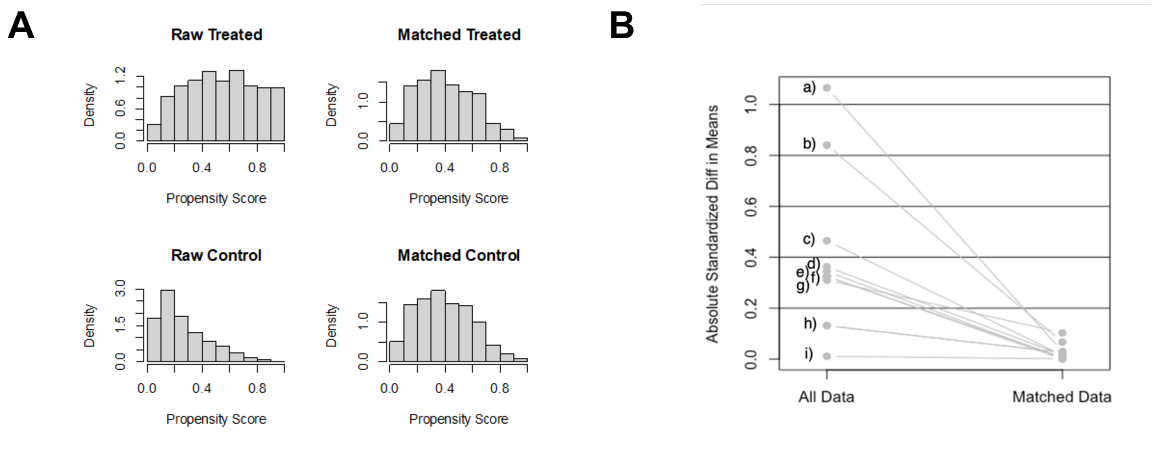

A) Propensity scores before and after matching. B) Standardized mean differences of variables before and after matching: a) age, b) origin MSBase*, c) EDSS, d) relapses before baseline, e) disease duration, f) origin Swedish MS registry*, g) MSSS, h) sex, i) origin Italian MS registry*

EDSS = Expanded Disability Status Scale, MSSS = Multiple Sclerosis Severity Scale, *OFSEP used as reference value.

List of R packages used for the statistical analysis

- tableone
- gdata
- zoo
- dplyr
- lsr
- MatchIt
- pscl
- survival
- survminer
- ggplot
